# Supplementary material for: Novel Dual PI3K/mTOR Inhibitor, Apitolisib (GDC-0980), Inhibits Growth and Induces Apoptosis in Human Glioblastoma Cells
Source: Int J Mol Sci. 2021 Oct 26;22(21):11511. doi: 10.3390/ijms222111511 (PMC8583746; doi:10.3390/ijms222111511)
Supplement: Supplementary file 1 [file ijms-22-11511-s001.zip › ijms-1408547-supplementary.pdf]

Supplementary Materials

Table S1. The ratio between the means of densitometric values of P-EIF2α /total EIF2α.

A172			U118			
**Control**	**10 μM**	**20 μM**	**Control**	**10 μM**	**20 μM**	
0.187590	0.229013	0.302291	0.703806	0.555065	0.818986	**EIF 2α**
0.252652	0.288993	0.305706	0.562119	0.380853	0.213949	**P-EIF 2α**
**1.343**	**1.262**	**1.011**	**0.799**	**0.686**	**0.261**	**P-EIF 2α/ EIF 2α**
